# Enhancing diabetes self-management through the AADE7 self-care behaviors framework: an observational study

**DOI:** 10.31744/einstein_journal/2025AO1213

**Published:** 2025-02-14

**Authors:** Denise Machado Mourão, Gean Moreira Silva Santos, Glaucia Cruzes Duarte

**Affiliations:** 1 Universidade Federal do Sul da Bahia Teixeira de Freitas BA Brazil Universidade Federal do Sul da Bahia, Teixeira de Freitas, BA, Brazil.; 2 Universidade Federal de São Paulo São Paulo SP Brazil Universidade Federal de São Paulo, São Paulo, SP, Brazil.

**Keywords:** Diabetes, Patient education, Self-care, Self management, Patient-centered care, Family support, Blood glucose, Insulin, regular, human

## Abstract

**Objective:**

We aimed to implement a comprehensive and accessible diabetes education program for patients and their families based on the AADE7 framework to enhance diabetes self-management and clinical outcomes.

**Methods:**

An observational, descriptive, mixed-methods study was conducted using weekly online webinars to deliver AADE7 educational content to participant groups. Data collection involved pre and postintervention assessments of knowledge and participant feedback.

**Results:**

A total of 123 participants (72 with diabetes, 5 with prediabetes, and 46 family members) from various Brazilian regions participated in the study. The majority of family members were female and from low-income backgrounds. Most participants had limited prior diabetes education. The educational sessions improved participants’ understanding of diabetes management, including the importance of monitoring blood glucose, administering medication, managing diet, and maintaining physical activity. The program also addressed common misconceptions, including managing insulin types, preventing lipodystrophy, adequately correcting hypoglycemia, and incorporating sweets and other carbohydrate-rich foods into the diet when appropriate.

**Conclusion:**

Diabetes education enhances glycemic management; therefore, it should be an integral part of treatment. The AADE7 framework proved highly effective, offering a holistic perspective on diabetes care.

## In Brief

Participants had limited exposure to prior diabetes education but improved their understanding of key components of diabetes management, including glucose monitoring, medication, diet, and exercise. Misconceptions regarding insulin use, lipodystrophy prevention, hypoglycemia correction, and appropriate consumption of snacks and carbohydrates were effectively addressed.

## Highlights

■ Diabetes education is the central pillar of treatment, and the AADE7 offers a 360-degree view of care.

■ Only 18.9% of participants had previously engaged in diabetes-related educational programs.

■ Recognizing signs of hypo and hyperglycemia and responding appropriately is crucial to avoiding complications.

■ Diabetes and sugar consumption remain taboo for many, including healthcare professionals.

## Graphical abstract

**Figure:**
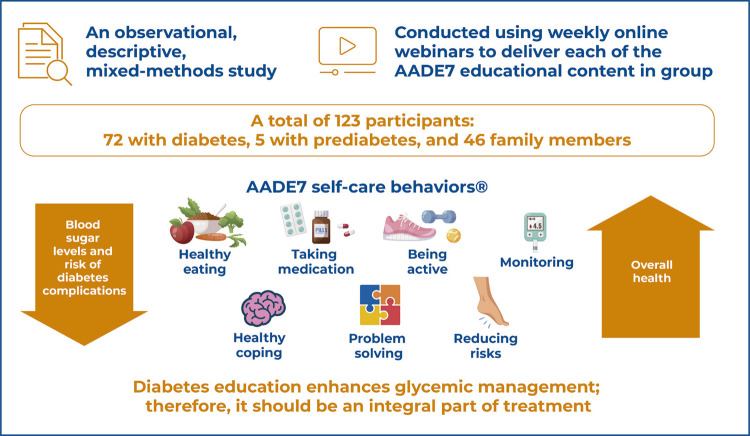

## INTRODUCTION

Diabetes is one of the most serious, costly, and rapidly growing chronic health conditions worldwide.^1^ Since the onset of the COVID-19 pandemic, the prevalence of diabetes has risen significantly,^2-4^ turning it into a major challenge for health systems globally.

After receiving a diagnosis, individuals with diabetes and their families face an urgent need to change their routines, requiring substantial support to learn and develop self-care behaviors. Self-care education should be provided-and updated at least annually-in all cases. It is a necessary support tool when treatment targets are not achieved, physical or psychosocial complications arise, or during care transitions.^5^

Diabetes self-management education is considered the cornerstone of other pillars of treatment, including medication, nutrition therapy, and physical activity. This concept emphasizes an empowerment-based approach to treatment.^5^ Without adequate education, patients struggle to achieve sustained success in their care, making them more vulnerable to micro-and macrovascular complications. In this context, the AADE7 Self-Care Behaviors^®^ (AADE7) framework has been recognized as a comprehensive and effective tool for diabetes self-management. It promotes behavioral changes, effective self-management and improved clinical outcomes.^6^

However, numerous studies have highlighted the vulnerability of patients who lack access to adequate diabetes education programs. These challenges often stem from insufficient knowledge and limited access to trained multidisciplinary teams.^7-10^

## OBJECTIVE

To implement a comprehensive and accessible education program for people with diabetes and their family members, based on the AADE7 framework, to enhance diabetes self-management and improve clinical outcomes.

## METHODS

### Research design, ethical aspects, and participants

This was an observational, descriptive, mixed-methods study reporting on the experience of a group educational intervention conducted via webinars based on the AADE7 framework.^6^ The study was approved by the *Universidade Federal do Sul da Bahia* (UFSB) Research Ethics Committee (CAAE: 53423421400008467; # 5317202).

Individuals with diabetes or prediabetes, as well as family members of Individuals with diabetes, were invited to participate via social media posts from the Reference Center of Diabetes in Schools of Teixeira de Freitas/BA (CRDE-TxF) in Brazil. Additionally, snowball sampling was employed to recruit more participants.^11^

Inclusion criteria were as follows: individuals with diabetes or prediabetes or those caring for a family member with diabetes. Exclusion criteria were as follows: the absence of a smartphone or computer with Internet access required to participate in the educational sessions.

### Procedures and analysis

All participants first signed a Consent Form and completed a registration form that collected personal data and information regarding their diabetes treatment. The registration form provided a brief explanation of each AADE7 behavior and asked participants to identify areas where they had the least knowledge. After completing the forms, participants received a schedule and a link for the educational group meetings. The intervention consisted of weekly 90-minute online meetings over 2 months, covering the following topics: 1) Monitoring: importance of blood monitoring; care and maintenance of glucometers and measuring strips; common errors in measurements; proper disposal of lancets and strips; frequency and timing of measurements; interpretating blood glucose results; the importance of maintaining a thorough glycemic diary, and advantages of continuous monitoring sensors; 2) Taking medication: oral medications for T2D; types of insulins and their actions (onsets, peaks, durations); proper insulin administration using syringes or pens; storage, transport and disposal of insulin devices; 3) Healthy eating: myths and truths about the diabetic diet; macronutrients and different types of carbohydrates; impact of macronutrients on blood glucose; glycemic index and load and nutritional strategies; portion sizes; reading and interpreting food labels; clarifications about foods labeled as diet or light; using MyPlate and the 10 steps to healthy eating; carbohydrate count; 4) Being active: benefits of physical activity; reducing sedentary time; exercise types and their effects on blood glucose; age-appropriate duration and frequency of physical activity; home-based exercise strategies; 5) Problem solving: definition and symptoms of hypoglycemia and hyperglycemia; identifying different levels of hypoglycemia and adjusting the actions to be taken at each case; possible causes of hypoglycemia; diabetes kit; the importance of always carrying a personal identification about having diabetes; 6) Reducing risks: preventing complications of diabetes (*e.g.*, retinopathy, kidney disease, neuropathies, foot ulcer or infection, dyslipidemia, cardiovascular disease, stroke, periodontal disease) and the importance of immunization in people with diabetes; and 7) Healthy coping: five stages of grief after diabetes diagnosis according Kübler-Ross, managing diabetes-related distress and support networks; managing glycemic alterations resulting from lifestyle factors (the intake of fats, proteins and alcohol; tobacco, puberty, menstrual period, sleep, dawn phenomenon and Somogyi effect, celiac disease; sick days and medications; excessive exposure to the sun; parties, trips, use of technology, the rights of people with diabetes, diabetes in schools).

Google Meet was used for the meetings, and participants were encouraged to speak voluntarily, share experiences, and provide feedback. Data on prior knowledge or diabetes management skills were collected and expressed as attendance rates (percentages) or means (M) with standard deviations (SD) for categorical and continuous variables, respectively.

The CRDE-TxF team, comprising professors and students from the *Faculdade de Medicina, Universidade Federal do Sul da Bahia*, was responsible for all processes, including recruitment, development and implementation of the intervention content.

## RESULTS

### Patient characteristics

**Figure 1:**
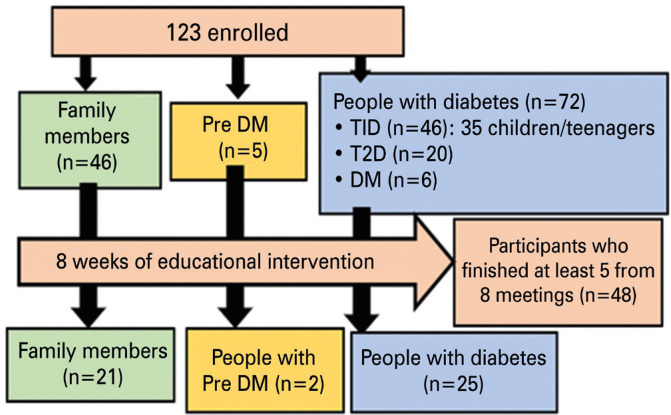
Sample and methodological pathway

A total of 123 participants were enrolled in the study: 72 had diabetes, five had prediabetes, and 46 were family members of individuals with diabetes (Figure 1). Participants were distributed across four distinct Brazilian regions: 56.9% from the Northeast, 37.4% from the Southeast, 4.1% from the North, and 1.6% from the Mid-west. Only 18.9% of participants had previously participated in any type of diabetes education activity. The family members group (n=46) age ranged 21-58 years, with a mean age of 40.8+8.5 years. The majority of family members (91.3%) were female. Regarding education levels, 54.3% had completed high school level, 34.8% had university-level education, and 10.9% had only middle school education. In terms of household income, 67.4% were from low-income households, while 19.6% belonged to low-middle-income households, and 6.5% each were from upper-middle-income and high-income households.

**Figure 2:**
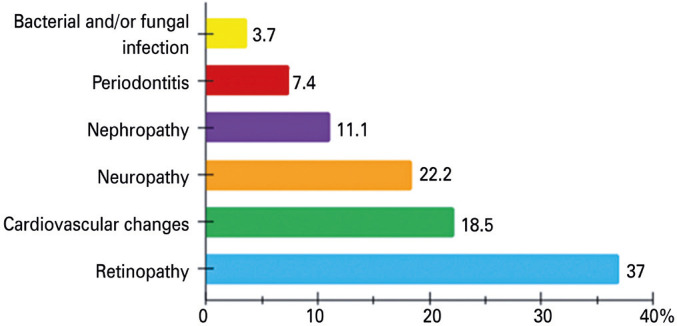
Types and frequency of complications in participants with diabetes

Among the participants with diabetes (Table 1), 47 had type 1 (T1DM), 20 had type 2 (T2DM), and five were unsure of their diabetes type or reported it incorrectly. For example, two participants claimed to have T1DM but had never used insulin. Of the 72 enrolled patients with diabetes, 15 reported having complications (Figure 2).

**Table 1:** Characteristics of participants with type 1 diabetes and type 2

Diabetes type	T1DM (n=47)		T2DM (n=20)	
	Range	n (%)	Range	n (%)
Age (years)				
	<10	10 (21.3)	18-34	1 (5)
	10-18	25 (53.2)	35-60	12 (60)
	>18	12 (25.5)	> 60	7 (35)
Diagnostic time (years)				
	<1	13 (27.6)	<5	6 (30)
	1.1-5	17 (36.2)	5-10	8 (40)
	5.1-10	7 (14.9)	>10	6 (30)
	>10	10 (21.3)		
A1c (%)	<7	6 (12.8)	<7	2 (10)
	7-8.4	18 (38.3)	7-8.4	2 (10)
	>8.5	13 (27.6)	>8.5	6 (30)
	NI	10 (21.3)	NI	10 (50)
BMI (kg/m^2^)	18.5-24.9	29 (61.8)	18.5-24.9	3 (15)
	25-29.9	1 (2.1)	25-29.9	2 (10)
	30-34.9	0 (0)	30-34.9	4 (20)
	35-9.9	1 (2.1)	35-39.9	0 (0)
	>40	0 (0)	>40	2 (10)
	NI	16 (34)	NI	9 (45)

BMI: body mass index; NI: not informed.

Out of the 11 participants with T2DM who provided their weight and height, 10, 20, and 15% had a body mass index (BMI) >40 or 25-29.9kg/m^2^, 30-34.9kg/m^2^, and <25kg/m^2^, respectively, all of whom were adults. Among those with T1DM, 31 provided data on BMI, of which 29 were children or teenagers. In this group, 64% had a BMI below 25, while 2.1% had a BMI between 25 and 29.9kg/m^2^.

Approximately 47 participants reported their A1c levels before the study, and only 17% (n=6 T1DM and n=2 T2DM) had an A1c below 7%.

Only 3 out of 72 participants with diabetes used continuous glucose monitoring systems. Among the 53 participants using insulin, 79.3% used pens, 5.7% used syringes (including two women aged >60 years with T2DM and low vision), 7.5% used insulin pumps, and 7.5% did not specify their chosen devices. Regarding the type of insulin used, 62.2% used insulin analogs, 18.9% used human insulin, and 18.9% did not report the type of insulin used.

The two women with T2DM and low vision mentioned reported receiving only 25 syringes (graduated syringes with a capacity of up to 100 U) every two months despite being on basal-bolus therapy with irregularly prescribed doses.

Among the AADE7, Healthy Eating and Healthy Coping were the areas where participants expressed the most uncertainty, with response rates of 60 and 57, respectively.

At the end of each meeting, participants shared impactful reflections on their experiences. Below are selected excerpts, organized by AADE7 topics:

Monitoring: “What most caught my attention in this meeting was the importance of showing accurate values in the glycemic diary for the doctor” (15-year-old girl with T1DM). “Pricking the side of the finger instead of the center to get a drop of blood was new for me”; “I learned that there is no point in using strips after they expire”; “I used to throw them (lancets and strips) in the regular trash”; “The highlights of this meeting were about glucose levels interpretation (glycemic targets) and how helpful it is to keep a good record of them for fine adjustments and more precise prescriptions.”

Taking medication: “I had never heard of the need to homogenize the neutral protamine hagedorn (NPH) insulin 20 times before use” (Mother of a T1DM teenager and 38-year-old man with T1DM for 20 years and advanced retinopathy); “I did not know different insulins had different peaks and time durations… I thought they were all the same” (mother of a 25-year-old man diagnosed with T1DM for 13 years); “I did not know we should check needle flow before using (the insulin pen) and to not inject cold insulin” (mother of a 17-year-old girl with T1DM for 5 years). “She only takes her insulin shots in her tummy … she says in other parts it is too painful. This may be because she uses it cold and does not change the needle, as you taught today” (mother of a 10-year-old girl with 5 years of T1DM, after learning about lipodystrophy and the importance of rotating injection sites). “Once I took Regular shot instead of the NPH morning dose, when I was still working. I had huge hypoglycemia with fainting and everything else. They took me to the hospital, and I lost my job.” (48-year-old woman with T1DM, on the importance of making a very distinct mark in both insulin pens to tell the difference between Regular and NPH); “I did not know we should pay attention to the expiry date after starting insulin use, instead of the one on the label” (Mother of a child with T1DM).

Healthy eating: “I was taught a diabetic person cannot eat anything with sugar - sweets’’ (Mother of a 5-year-old girl with T1DM and a 25-year-old woman with T1DM); “As soon as the diabetes started when he was 3-years-old, we kept him from many foods, until years later we learned about counting carbs.” (Mother of a 17-year-old boy with T1DM).

Being active: “I even took her out of volleyball because I thought it was bad for her… her gym teacher said she always looked tired, and her pediatrician had not instructed me about that … At that time, I kept her glucose levels between 70 and 90” (Mother of 11-year-old girl with T1DM).

Problem-solving: Approximately 84.7% of participants did not know the three levels of hypoglycemia or the appropriate actions for each, but many recognized their symptoms: “I feel a lot of tingling in my nose” (24-year-old man with T1DM); “cold sweats and shaking (22- and 15-year-old men with T1DM); “My son gets sunken eyes and pallor” (Mother of 6-year-old boy with T1DM); “I freak out just thinking it could happen overnight… I never sleep well.” (Mother of a 3-year-old boy with T1DM). Hyperglycemia symptoms: “She/he feels tummy ache” (mothers of 3- and 6-year-old boys with T1DM). “I feel very tired, sleepful, and very stressed” (15-year-old girl with T1DM). “I had never heard about the honeymoon phase before her second ketoacidosis hospitalization” (Mother of a 15-year-old girl with T1DM); “sometimes I feel lost with the bolus corrections, because 2 hours later his glucose level is still high” (Mother of an 8-year-old boy with T1DM).

Reducing risks: “Are diabetes complications more related to hypo or hyperglycemia? How can they be avoided?” (A 58-year-old man with T2DM). “I never had my feet examined when I got to the endocrinologist … and I pay for those appointments… he also forgets to ask me to do my eye exam, which I learned here should be done annually, too.” (24-year-old woman with 11 years of T1DM after learning about foot care and the checklist exams); “I play a lot of football barefoot” (14-year-old boy with T1DM).

Healthy coping: About the five stages of grief “My mother is still in the bargaining phase, I have had diabetes for 11 years and she still thinks if I do something I will get better…” (24-year-old woman with T1DM); “After diabetes, everyone in our family was afraid to take care of him, even his grandma... now only his dad and I do that. Sometimes he would stay with my brother, but they let him go hungry” (mother of a 3-year-old boy with T1DM).

On other factors affecting glycemia: “In my premenstrual period it is more difficult to keep blood sugar control,” “for me, it’s in the menstrual days” (15- and 16-year-old girls with T1DM); “When I get stressed, it gets higher” (18- and 38-year-old men with T1DM); “In sick days, my glycemia goes to the heights” (15-year-old girl with T1DM), “here, in case of a flu or other infections it’s nerve-racking, because even with quick insulin correction shots, the glycemia does not decline,” “Viruses also increase glycemia a lot” (mothers of girls with T1DM), “Sick days, stress… when I had COVID it was terrible to get glucose control… I did not know how much more insulin to take… contraceptives can also have interactions...” (24-year-old woman with T1DM).

By the end of the educational sessions, most participants expressed feeling more confident in managing diabetes self-care due to their newly acquired knowledge.

## DISCUSSION

The majority of participants in the family member group were female, which corroborates findings from previous studies indicating that women are often more involved in the care of people with diabetes.^12-14^ Sharing diabetes care responsibilities among family members is crucial, particularly for children and teenagers with T1DM. In this context, support from diabetes educators’ is important to help families avoid distress and poor glycemic control.^15,16^

Another notable characteristic of our sample was that nearly one-third of participants with T1DM had been diagnosed within the past year, suggesting that many were likely experiencing the honeymoon phase. Also known as the partial remission phase, this period is characterized by residual pancreatic beta-cell functionality of a small portion of the remaining pancreatic beta cells, temporarily reducing the need for exogenous insulin.^17^ During this phase, diabetes education and frequent insulin dose adjustment are critical to prevent ketoacidosis, as highlighted by the mother of a T1DM teenage participant.

In Brazil, many people with diabetes lack access to a multidisciplinary healthcare team or sufficient endocrinologist appointments,^18^ compromising their treatment. Our data reflected this, as most participants had never participated in a diabetes education program, underscoring Brazil’s limited availability of free diabetes education initiatives.

Participants displayed a range of misconceptions about basic concepts, including their diabetes types. For example, some participants identified as having T1DM despite never using insulin. Others were unaware of critical practices, such as homogenizing NPH insulin before use, not using expired strips, and properly disposing of diabetes supplies. Another aspect that needed emphasis was clearly and visibly marking insulin pens to distinguish between different types of insulin, such as regular and NPH. In Brazil, both types of pens are designed with similar colors, differing only in a small color detail on the top of the ejector bottom. This similarity increases the risk of patients confusing the pens and administering the wrong insulin.

An important topic with which the participants expressed unfamiliarity was the proper steps to avoid lipodystrophy during insulin administration. These steps include the following: 1) use a new 4-mm needle for each injection; 2) inspect and palpate the injection site before administering insulin; 3) rotate injection sites properly-recommended areas include the abdomen (avoiding a two-centimeter radius around the navel), outer thighs (maintaining at least one hand’s distance from the knee or hip), upper outer arms, upper outer glutes, and the flanks.

To prevent lipodystrophy, patients should avoid injecting in the same spot for at least 14 days and ensure there is a minimum distance of 1cm between successive injections.^19,20^

In the context of diabetes education, practical aspects of diabetes care that intersect with the patient’s social life, such as diet and exercise, must be considered. Unfortunately, some health professionals continue to perpetuate “carbophobia”^21^ -the unfounded fear of carbohydrates-rather than promoting carbohydrate counting, a well-established and evidence-based nutritional strategy for diabetes management.^22^

Physical activity and exercise are fundamental pillars of diabetes treatment,^5,23^ as they contribute to a reduced need for antihyperglycemic agents and insulin.^24^ However, a lack of understanding among teachers and parents about the role of exercise in glycemic control persists. As a result, some children and adolescents with diabetes are still excluded from certain physical activities in schools.^25^ The primary cause of this exclusion is the fear of hypoglycemia, a concern that can be addressed through diabetes education, which serves as a critical tool for dispelling misconceptions and preventing the perpetuation of these poorly understood concepts.^26^

As shown in the literature^27,28^ and observed in our study, retinopathy was the most frequent complication reported among patients with poor glycemic control. Therefore, annual screening through fundoscopy should be encouraged, beginning at the time of T2DM diagnosis and 5 years after the onset of T1DM.^29^

Education on proper foot care for people with diabetes is another critical area of concern, as it helps prevent injuries, foot ulcers and amputations.^30,31^ This is particularly important since 50% of diabetic peripheral neuropathy cases may be asymptomatic.^29^ In this study, neuropathy was identified as the third most common complication, following cardiovascular changes and retinopathy, although its prevalence was likely underestimated.

A yearly comprehensive foot exam by a trained health professional is recommended.^25^ Additionally, patients or caregivers should take daily practical measures to prevent foot complications, including 1) inspecting feet daily, 2) avoiding moisture between toes, 3) choosing white cotton socks without elastics, 4) cutting toenails straight rather than rounded, 5) hydrating the skin appropriately, and 6) wearing comfortable shoes with nonslip soles to protect the feet.^32^

In Brazil, most individuals with diabetes receive human insulin and treatment supplies from the government. However, the quantity of supplies-such as lancets, strips, syringes, and pen needles-is often insufficient, compromising adequate glycemic control.^33,34^ Furthermore, regular educational programs to support people with diabetes and their families are largely unavailable across the country.

In our study, most insulin users reported using insulin analogs in pens, even though both analogs and human insulins are available in pen and syringe forms. Human insulins, typically distributed in syringes, are the most commonly provided form. The higher usage of analog insulin pens among participants can be explained by the fact that the majority of these users were children or adolescents with T1DM, who, by law, have greater access to analogs in pens.

### Strengths and limitations of the study

The strengths of this study are twofold. First, it provides a comprehensive and detailed methodology for implementing the AADE7 framework, covering all aspects of diabetes care and establishing a structured diabetes education program. Second, the study’s virtual nature expanded its reach, enabling participation from individuals in smaller towns and across four of Brazil’s five states, particularly in regions with limited access to diabetes education and professional guidance.

However, the study also had several limitations. The remote format excluded individuals in situations of high social vulnerability, such as those facing illiteracy or lacking access to smartphones, computers or a stable internet connection. As observed in other studies with longer interventions, many participants were unable to attend at least 5 of the 8 proposed educational sessions due to the limitations of virtual delivery. Despite the encouraging testimonials shared by participants during and after the sessions regarding the importance of the knowledge gained, there is no guarantee that participants will implement all they were taught in their daily lives.

## CONCLUSION

Monitoring, support, and continuous follow-up using education as a pedagogical tool in multidisciplinary interventions are essential for preventing the acute and chronic complications of *diabetes mellitus*. The implementation of Diabetes Education Programs, as proposed in this study, has proven effective. It enhances participants’ communication and listening skills while allowing the multidisciplinary team to assess patients’ understanding of their disease in real-time, identify their actual needs, and involve them and their families in the diabetes care process. Feedback after the weekly online meetings indicated an improvement in their understanding of diabetes education concepts. This enhanced understanding motivated lifestyle changes, improved self-management routines, and encouraged acceptance of living with diabetes within their familial and social contexts. Reviewing AADE7 themes stimulated learning and empowered participants to take a proactive role in managing their condition.
